# Early somatosensory processing in individuals at risk for developing psychoses

**DOI:** 10.3389/fnbeh.2014.00308

**Published:** 2014-09-11

**Authors:** Florence Hagenmuller, Karsten Heekeren, Anastasia Theodoridou, Susanne Walitza, Helene Haker, Wulf Rössler, Wolfram Kawohl

**Affiliations:** ^1^The Zurich Program for Sustainable Development of Mental Health Services (ZInEP), Psychiatric Hospital, University of ZurichZurich, Switzerland; ^2^Department of Psychiatry, Psychotherapy and Psychosomatics, Psychiatric Hospital, University of ZurichZurich, Switzerland; ^3^Department of Child and Adolescent Psychiatry, University of ZurichZurich, Switzerland; ^4^Translational Neuromodeling Unit, Institute for Biomedical Engineering, University of Zurich and ETH ZurichZurich, Switzerland; ^5^Laboratory of Neuroscience (LIM 27), Institute of Psychiatry, University of Sao PauloSao Paulo, Brazil

**Keywords:** somatosensory evoked potentials, schizophrenia, high-frequency oscillations, risk, thalamus dysfunction, cannabis

## Abstract

Human cortical somatosensory evoked potentials (SEPs) allow an accurate investigation of thalamocortical and early cortical processing. SEPs reveal a burst of superimposed early (N20) high-frequency oscillations around 600 Hz. Previous studies reported alterations of SEPs in patients with schizophrenia. This study addresses the question whether those alterations are also observable in populations at risk for developing schizophrenia or bipolar disorders. To our knowledge to date, this is the first study investigating SEPs in a population at risk for developing psychoses. Median nerve SEPs were investigated using multichannel EEG in individuals at risk for developing bipolar disorders (*n* = 25), individuals with high-risk status (*n* = 59) and ultra-high-risk status for schizophrenia (*n* = 73) and a gender and age-matched control group (*n* = 45). Strengths and latencies of low- and high-frequency components as estimated by dipole source analysis were compared between groups. Low- and high-frequency source activity was reduced in both groups at risk for schizophrenia, in comparison to the group at risk for bipolar disorders. HFO amplitudes were also significant reduced in subjects with high-risk status for schizophrenia compared to healthy controls. These differences were accentuated among cannabis non-users. Reduced N20 source strengths were related to higher positive symptom load. These results suggest that the risk for schizophrenia, in contrast to bipolar disorders, may involve an impairment of early cerebral somatosensory processing. Neurophysiologic alterations in schizophrenia precede the onset of initial psychotic episode and may serve as indicator of vulnerability for developing schizophrenia.

## Introduction

Schizophrenia and bipolar disorders have been considered as two distinct disorders since Emil Kraepelin (1856–1926) divided psychotic illness into two diagnostic categories (Craddock and Owen, 2005). However, schizophrenia and bipolar disorders have a number of clinical and epidemiological features in common. Following Griesingers (1817–1868) unitary concept of psychosis called “Einheitspsychose,” it has been suggested that psychotic symptoms may be distributed along a continuum (Crow, 1986). To date it is still unclear whether or not these two major psychoses are distinct entities or if bipolar disorder and schizophrenia patients share, e.g., common neurophysiologic dysfunction (Alaerts and Del-Favero, 2009; Kurnianingsih et al., 2011; Whalley et al., 2012; Redpath et al., 2013). Both distinctive and similar patterns of brain structural abnormality were observed in patients with schizophrenia vs. bipolar disorders (McDonald et al., 2004; Murray et al., 2004).

The thalamus is considered to play a crucial role in the pathophysiology of psychiatric illnesses such as schizophrenia (Andreasen, 1997) and mood disorders (Soares and Mann, 1997). The thalamus forms a variable gate of access for sensory information to reach the cerebral cortex (McCormick and Bal, 1994). Dysfunctional cortico-striato-thalamic connections and abnormal thalamocortical connections associated with schizophrenia were widely demonstrated (Carlsson and Carlsson, 1990; Andreasen, 1997; Jones, 1997; Woodward et al., 2012). Furthermore, schizophrenic patients show anatomic (Andreasen et al., 1994; Gur et al., 1998; Portas et al., 1998; Staal et al., 1998; Hazlett et al., 1999; Ettinger et al., 2007) and metabolic (Szechtman et al., 1988; Siegel et al., 1993; Buchsbaum et al., 1996; Holcomb et al., 1996; Kim et al., 2000) thalamic alterations. In contrast to this, no abnormalities in thalamic size have been found in persons with bipolar or unipolar affective disorders (Caetano et al., 2001; Mamah et al., 2010). Nonetheless, functional and neurochemical abnormalities in this brain region have been reported for individuals with bipolar disorders (Buchsbaum et al., 1997; Deicken et al., 2000).

Alterations of preattentive sensory gating as reported in neurophysiological studies emphasize the central role of the thalamus in the regulation of cortical input. The prepulse inhibition has been shown to be abnormal in patients with schizophrenia and their relatives (for a review, see Braff, 2010). The findings are not equally consistent for patients with bipolar disorder (Thaker, 2008). However, two studies reported an abnormal prepulse inhibition in patients with bipolar disorder (Perry et al., 2001) and their first-degree relatives (Giakoumaki et al., 2007), whereas Carroll et al. (2007) found no difference in prepulse inhibition between bipolar patients and healthy controls.

Despite their advantageous high temporal resolution, electrophysiological studies of possible thalamocortical dysfunction in schizophrenia or bipolar disorder other than the startle response are rare. Later evoked potential phenomena have been studied more often than early ones, i.e., occurring within 50 ms after stimulus application (Shagass et al., 1977). However, early evoked potentials are less susceptible to changes by uncontrollable factors such as, e.g., attention, and their underlying neurophysiology is better understood than that of the later potentials (Shagass et al., 1977; Buchner et al., 1995).

Thalamocortical and early cortical processing can be investigated accurately with advanced analysis of human median nerve somatosensory evoked potentials (SEPs) (Shagass et al., 1977; Lehtonen, 1981; Buchner et al., 1995): SEPs of the median nerve show a brief oscillatory burst with low amplitudes (<500 nV) and high frequency (~600 Hz) which can be isolated with high-pass filtering. These high-frequency oscillations (HFOs) underlie the primary cortical low-frequency responses represented by a parietal negativity peak component ~20 ms after stimulation, i.e., the N20 (Yamada et al., 1988). Functional dissociation of these two evoked responses indicates different origins for the low- and high-frequency SEP-components (Emerson et al., 1988; Yamada et al., 1988; Klostermann et al., 1998, 1999; Hashimoto et al., 1999; Gobbele et al., 2000, 2004; Halboni et al., 2000). The N20 is mainly generated by excitatory postsynaptic potentials in Brodman area 3b pyramidal cells (Allison et al., 1991). The generators of HFO have been proposed as the thalamus, the thalamocortical fibers or postsynaptic activities in the primary sensory cortex (for a review see Curio, 2000). Early and late HFOs, i.e., the wavelets before and after the peak latency of the N20 as the initial cortical response, have been supposed to be separate since these two components differ in their responsiveness to various modulations (Ozaki et al., 2001; Ritter et al., 2008). The early part of the HFOs is presumably generated from action potentials of thalamocortical fibers (Klostermann et al., 1999; Curio, 2000; Gobbele et al., 2004). There remains a controversy on the generation of the later part of somatosensory HFOs. Pyramidal “chattering” cells (Gray and McCormick, 1996), cortical fast-spiking inhibitory interneurons (Hashimoto et al., 1996) and thalamocortical relay cells (Curio, 2000) have been proposed as possible cell populations generating HFO. Supporters of the interneuron hypothesis propose that the late HFO represent the activities of combined vertically and horizontally oriented GABAergic inhibitory interneurons in somatosensory cortex (Hashimoto et al., 1996; Ozaki et al., 2001; Ozaki and Hashimoto, 2005, 2011). They rely on the divergence in the orientation of the dipoles between HFO source and underlying N20 source (Ozaki et al., 2001; Ozaki and Hashimoto, 2011).

Previously, the variability of wave shapes of SEPs in chronic schizophrenic patients was described as a high early and low late stability of amplitudes (Shagass et al., 1977; Lehtonen, 1981) probably depending on the acuity of the disease. Norra et al. (2004) reported alterations of the thalamocortical somatosensory signal processing in schizophrenia. To our knowledge, no such study exists for bipolar disorder or for subjects at risk of schizophrenia or bipolar disorder yet.

This non-invasive electrophysiological study aims to explore potential differences in early sensory filtering in at risk probands for schizophrenia and bipolar disorder by analyzing low-frequency responses and HFOs in relationship to the symptoms. We hypothesize that alterations of signal processing observed in clinical populations are observable also at the at-risk level in a way comparable to the findings of Norra et al. (2004). In that study later HFOs in subjects with schizophrenia and higher N20 dipole source strengths were reported. The later HFOs are interpreted as epiphenomena of poorer thalamocortical filtering and the larger N20 as epiphenomena of a larger signal strength following this poorer filtering. The analysis was performed with regards to cannabis use as it is considered to increase the risk of developing psychosis (Kawohl and Rössler, 2008; Roser et al., 2010; Gururajan et al., 2012).

## Material and methods

### Subjects

Subjects at risk were recruited in the Canton of Zurich, Switzerland, in the context of a prospective longitudinal multi-level-approach (psychopathology, neuropsychology, electrophysiology, sociophysiology, genetic, MRI) on early recognition of psychoses within the framework of the “Zurich Program for Sustainable Development of Mental Health Services” (Zürcher Impulsprogramm zur nachhaltigen Entwicklung der Psychiatrie, i.e., ZInEP, http://www.zinep.ch). The present study analyses data from the first step of assessment in a cross-sectional design. The recruitment of subjects was carried out through a study website, flyers and newspaper advertising, or the subjects were referred to the study center by general practitioners, school psychologists, counseling services, psychiatrists, or psychologists. Following an initial screening, diagnostic interviews were administered face-to-face. After complete description of the study to participants, written informed consent was obtained; in case of minors including the written informed consent of their parents. The study was approved by the regional ethics committee of the canton of Zurich and was in accordance with the Declaration of Helsinki. For assessment of psychopathological symptoms all participants of the study were examined carefully by clinically skilled psychiatrists and psychologists. As inclusion criterion for our study, subjects had to fulfill at least one of the following three criteria:

1. High-risk (HR) status for psychosis assessed by the adult (Schultze-Lutter et al., 2007) or children-youth (Schultze-Lutter and Koch, 2010) version of the Schizophrenia Proneness Interview (SPI-A/SPI-CY), with at least one cognitive-perceptive (COPER) basic symptom or at least two cognitive disturbances (COGDIS) basic symptoms.
2. Ultra-high-risk (UHR) status for psychosis as rated by the Structured Interview for Prodromal Syndromes (SIPS) (McGlashan et al., 2001) with at least one attenuated psychotic symptom, or at least one brief limited intermittent psychotic symptom, or a positive state-trait criterion (reduction in global assessment of functioning of >30% in the past year, plus either schizotypal personality disorder or first degree relative with psychosis).
3. At-risk state for bipolar disorder (at-risk-bip) was defined by a score ≥14 in the Hypomania Checklist (Angst et al., 2005), or a score of ≥12 on the Hamilton Depression Scale (Williams, 1988), or a first degree relative with a bipolar disorder and a reduction in global assessment of functioning of >30% in the past year.

The division into HR and UHR subjects within the schizophrenia-risk group was made with the aim to distinguish between subjects with a more general risk (HR) and subjects with imminent risk (UHR) of transition to manifest schizophrenia (Klosterkötter et al., 2011; Fusar-Poli et al., 2013).

In addition to the scales for early recognition, the PANSS (Kay et al., 1987) was conducted to measure also the more pronounced psychotic symptoms. PANSS scores are given in Table 1. Exclusion criteria for study participation were manifest schizophrenic, substance-induced or organic psychosis or bipolar disorder, current substance or alcohol dependence; age below 13 or above 35 years; or low intellectual abilities with IQ < 80. Among the 185 subjects with available HF-SEP data, 82 fulfilled the UHR criteria for psychosis (UHR group), 73 the HR criteria (HR group) and 30 only the at-risk for bipolar disorder criteria (at-risk-Bip group). Several subjects met two or all of the three inclusion criteria. In the UHR group, 71 also met the HR criteria and 8 the at-risk-Bip criteria. From the HR group, 69 subjects fulfilled also the at-risk-Bip criteria. Twenty eight subjects were removed from the analysis (9 from the UHR group, 14 from the HR group and 5 from the at-risk-Bip group) due to reduced data quality (10 subjects without signal, 18 subjects with low-level signal but without high-level signal). From the 157 subjects in a risk status included in analysis, 43 subjects met the three inclusion criteria, 71 subjects met two of the inclusion criteria (5 UHR and at-risk-Bip, 18 UHR and HR, 48 HR and at-risk-Bip) and 43 subjects met only one inclusion criterion (7 UHR, 11 HR and 25 at-risk-Bip). The at-risk-Bip group did not include HR or UHR subjects, the HR group no UHR subjects.

**Table 1:** **Sample descriptive**.

	***n***	**Sex**		**Age**		**Handedness**		**Medication**		IQ^*^	**PANSS^c^**		
		**w**	**m**	**Mean (sd)**	**Range**	**Right**	**Left**	**CPZe^a^*n* (mean)**	**AD^b^*n***		**Positive mean**	**Negative mean**	**General mean**
HC	45	19	26	20.7 (5.3)	13–35	39	6	0	0	109.1	–	–	–
Bip	25	10	15	23.5 (6.3)	14–35	23	2	2 (122.3)	9	109.4	8.8	11.7	26.5
HR	59	25	34	23.4 (5.2)	14–34	53	6	10 (115.0)	18	103.7	9.9	12.0	27.6
UHR	73	28	45	18.7 (4.8)	13–35	62	11	19 (184.6)	14	101.9	14.8	15.9	34.3

* *IQ was estimated from MWT-B for subjects >18 years and from LPS and HAWIK for subjects <18 years*.

a *Chlorpromazine equivalent*.

b *Antidepressants: HC, healthy controls; Bip, at-risk-bipolar; HR, high-risk; UHR, ultra-high-risk*.

c *Positive and negative symptom scale*.

Fifty healthy controls (HC), matched regarding age and gender rates to the whole at-risk group, were enrolled in the study, 45 of them with available HF-SEP data. The presence of any mental illness was excluded using the Mini-International Neuropsychiatric Interview (Sheehan et al., 1998). The UHR-group was significantly younger than the other groups (*p* < 0.005). The HC group and at-risk-Bip group had a significant higher IQ than the HR [*t*_(73)_ = 2.00, resp. *t*_(96)_ = 2.23, *p* < 0.05] and the UHR group [*t*_(88)_ = 2.29, resp. *t*_(111)_ = 2.79, *p* < 0.05]. There were no group difference in sex and handedness. When attenuated psychotic symptoms were associated with distress, some subjects were treated with antipsychotic medication. Antipsychotic medication status is given in chlorpromazine-equivalent (CPZe) dosage (Andreasen et al., 2010). Details are shown in Table 1.

### SEP recording

Subjects were requested to sit in a comfortable chair with their eyes open, in a quiet laboratory. They were instructed to relax and to avoid movements throughout the stimulus presentation sequence and the recording. Electrical transcutaneous stimulation was performed with two electrodes over the median nerve on the wrist of the dominant hand. Single constant-current square wave pulses of a duration of 0.2 s were delivered with an intensity of 4 mA above individual motor threshold (max. 20 mA) and a stimulus rate of 6 Hz. To preserve a stable level of vigilance during stimulus presentation, participants were asked to watch a “Mr. Bean” movie without sound. EEG data were recorded using a BrainAmp amplifier and the Brain Vision Recorder software (both Brain Products GmbH, Munich, Germany). Electrodes were applied to the scalp using carefully positioned nylon caps [BrainCap with 32 channels (Easycap, Herrsching-Breitbrunn, Germany)] in accordance with the international 10/20 system. Scalp electrode impedances were kept below 10 kΩ. EEG Channels were referenced to FCz. Data were collected with a sampling rate of 2500 Hz.

### Data analysis

Source reconstruction was performed individually for each subject with dipole source analysis applying the Brain Electrical Source Analysis (BESA 5.1.8: MEGIS, Munich, Germany; www.besa.de) software. Artifact-free sweeps containing 250 addresses over a period of 100 ms, from 20 ms before to 80 ms after the stimulation were included in the analysis. Single dipole sources were fitted for each subject for a time period between 14 and 24 ms. Although the optimal solution to model all the different early SEPs needs a complex source configuration with at least three dipoles (Buchner et al., 1995), for our aim to demonstrate differences in signal composition between the subgroups an approach with one dipole was considered to be sufficient. This is in accordance with other studies such as Norra et al. (2004) or Waberski et al. (2004). The resulting dipole waveform was digitally filtered. A low-pass filter of 450 Hz (12 dB/octave slope, zero phase shift) and a high-pass filter of 40 Hz (12 dB/octave slope, zero phase shift) were used to determine latency and strength of the low-frequency activity as estimated by dipole source analysis. The strength of the low-frequency activity source was determined semi-automatically (a) as the absolute value of the minimum of the source waveform between 14 and 24 ms (N20) and (b) as the N20 minus the value of the next positive peak (N20-P25). High-frequency filtering was done with a low-pass filter turned off and a high-pass filter of 450 Hz (12 dB/octave slope, zero phase shift) to extract HFOs. For early HFO-components peaking before the maximum of N20, latencies of the negative oscillatory maxima and maximum peak-to-peak amplitudes were measured. Latencies and amplitudes of the late HFO subcomponents were computed in the same way as for the early HFO burst peaking after the N20 maximum (Gobbele et al., 2003). All peaks were plotted with the software Python (Python Language Website, http://www.python.org) and inspected visually.

### Statistical analysis

All statistical tests were performed using the IBM SPSS Statistic 20.0 package for Windows (IBM Corp.). Visual inspection of histogram revealed normal distribution of data. The Kolmogorov–Smirnov-Test was omitted because of the large sample size. Levene-Test revealed that the variances were roughly equal, so that we used parametrical tests. Group comparisons were performed with Student's *t*-test or ANOVA and corrected for multiple comparisons with Games-Howell *post-hoc* test. Correlation coefficients for variables of SEPs and clinical psychopathological scores were calculated with Pearson's *r*. Statistical significance was taken as *p* < 0.05. Effect sizes (ES) were reported (Cohen's *d*, while *d* = 0.3 indicates a small, *d* = 0.5 a medium and *d* = 0.8 a large effect; and *r*-values, *r* = 0.1 indicating a small, *r* = 0.3 a medium and *r* = 0.5 a large effect).

## Results

### SEP parameters: group comparisons

Group differences were found for the strength of the N20 source as well as for amplitudes of the maxima of the early and of the late part of the HFO. Details are given in Table 2. *Post-hoc* tests revealed that, compared to the at-risk-bip group, N20 source activity were significantly lower in the HR [3.74, 95% CI (0.03–7.45), *p* < 0.05] and the UHR groups (3.90, 95% CI (0.26–7.54), *p* < 0.05). Furthermore, compared to the at-risk-bip group, amplitudes were significantly lower in the HR group for the early part of HFO [0.67, 95% CI (0.13–1.22), *p* < 0.05] and the late part of HFO [0.61, 95% CI (0.05–1.16), *p* < 0.05]. The HC group did not differ significantly from the risk groups.

**Table 2:** **SEP parameters for the different groups**.

		**HC *n* = 45**	**Bip *n* = 25**	**HR *n* = 59**	**UHR *n* = 73**	***F*^a^**	***ES* (r)**	***r*^b^**
N20	Strength^b^	−9.67 (4.4)	−12.42 (6.3)	−8.68 (4.2)	−8.53 (4.0)	5.23^***^	0.86	0.74
	Latency^c^	19.06 (1.5)	19.02 (1.5)	19.67 (1.3)	19.41 (1.3)	2.23		
N20-P25	Strength^b^	24.42 (9.2)	26.87 (11.1)	21.35 (8.3)	21.37 (8.7)	3.30^*^	0.21	0.04
HFO early	Amplitude^b^	1.58 (0.9)	1.86 (0.9)	1.19 (0.7)	1.32 (0.8)	4.91^***^	0.27	0.07
	Latency	17.90 (1.5)	18.05 (1.0)	18.27 (1.5)	18.08 (1.4)	0.60		
HFO late	Amplitude^b^	1.52 (0.9)	1.78 (0.9)	1.17 (0.6)	1.24 (0.7)	4.58^***^	0.26	0.07
	Latency	19.88 (1.7)	19.86 (0.9)	19.97 (3.0)	20.40 (1.4)	0.87		

a p < 0.05^*^, p < 0.005^***^.

b *in nAm*.

c *in ms. HC, healthy controls; Bip, at-risk-bipolar; HR, high-risk; UHR, ultra-high-risk; ES, effect size*.

### SEP parameters: group comparisons with regard to cannabis use

Subjects reporting several times a week, weekly and monthly use of cannabis were grouped together (because of small group sizes in the respective categories) to obtain the group “users,” as opposed to the group “non-users.” Individuals reporting rare use (less frequently than monthly) were removed from the comparison. The information about cannabis use of 37 subjects in the risk groups was not available. Details are given in Table 3. During SEP recordings the subjects did not experience acute cannabis effects, because they had not used cannabis on the day of examination. None of the subjects suffer from cannabis dependence, thus no withdrawal symptoms were observed during testing.

**Table 3:** **Cannabis-users vs. non-users split by group**.

		**HC *n* = 45**	**Bip *n* = 25**	**HR *n* = 59**	**UHR *n* = 73**	**Total *n* = 202**
Cannabis users	N	3	4	13	12	32
Cannabis non-users	N	35	11	32	30	108
Missing data	N		6	9	22	37
Rare cannabis use^*^	N	7	4	5	9	25

* *Was not included in the analysis; HC, healthy controls; Bip, at-risk-bipolar; HR, high-risk; UHR, ultra-high-risk*.

#### Comparison of source activity: cannabis users vs. cannabis non-users

**Figure 1:**
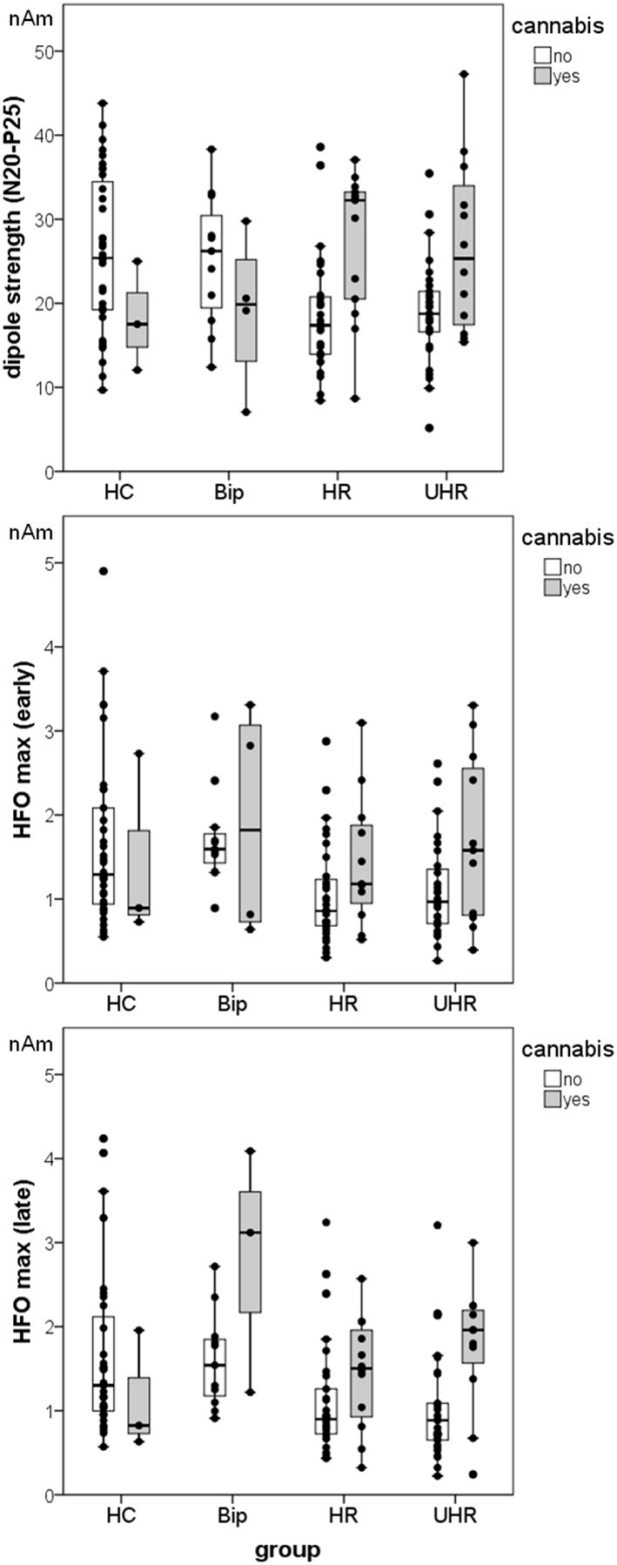
**Mean dipole source activity and distribution of data in the different groups, separated into cannabis users and non-users**. Note the small sample size of cannabis users in the Bip group (*n* = 4) and the HC group (*n* = 3). Vertical bars represent the SE.

In the at-risk-bip group, cannabis users showed slightly lower N20 strength than non-users, but this difference did not reach significance—but note the small sample size of users in this group (*n* = 4). Contrary to this, in both groups at risk for developing schizophrenia, cannabis users showed higher N20-P25 peak-to-peak source strength than non-users, in the HR group [*t*_(43)_ = 3.60, *p* < 0.005, *d* = 1.21], and the UHR group [*t*_(14.3)_ = 2.48, *p* < 0.05, *d* = 0.87]. Amplitudes of the high-frequency range were also higher in cannabis users than in non-users, but reached significance only for the late part of HFO in the UHR group [*t*_(38)_ = 3.06, *p* < 0.005, *d* = 1.12]. However, SEP source activity of cannabis non-users in the HC group did not differ significantly from those of cannabis users in the HR and UHR groups. Differences users vs. non-users in the HC group were not calculated because of only *n* = 3 cannabis users in this group. For details, see Figure 1.

#### Group comparisons among cannabis non-users

Among cannabis non-users, there were significant age differences between the groups [*F*_(3,104)_ = 8.72, *p* < 0.001], but the groups did not differ in sex. The HC group and the UHR group were significantly younger than both the at-risk-bip [*t*_(44)_ = −3.51, resp. *t*_(39)_ = −4.90, *p* < 0.005] and the HR groups [*t*_(65)_ = −2.73, resp. *t*_(60)_ = −3.66, *p* < 0.01]. Age did not correlate with dipole strengths, but correlated positively with the latencies of N20 (*r* = 0.201, *p* < 0.05) and of the early part of HFO (*r* = 0.199, *p* < 0.05) over the whole group of cannabis non-users. Antipsychotic medication estimated by CPZe dose (*n* = 31) did not correlate significantly with SEP parameters. In the UHR group however, subjects taking antipsychotic medication had lower early HFO amplitudes than those who did not take any medication [*t*_(48.2)_ = 3.20, *p* < 0.005].

**Figure 2:**
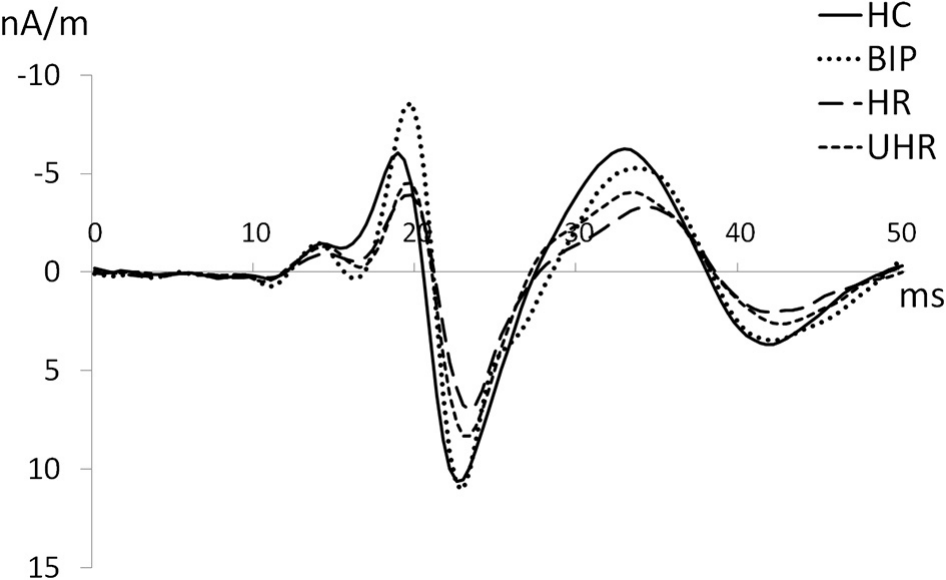
**Grand averages of N20 in the groups (40–450 Hz) among cannabis non-users**.

There was a significant effect of group on the N20 dipole strength [*F*_(3,104)_ = 4.27, *p* < 0.010, *r* = 0.33] and on the N20-P25 peak-to-peak strength [*F*_(3,104)_ = 7.56, *p* < 0.001, *r* = 0.42]. *Post-hoc* tests revealed that, compared to the HC group, N20 strengths resp. N20-P25 peak-to-peak strength were significantly lower in the HR [2.84, 95% CI (0.04–5.64), *p* < 0.05 resp. 7.46, 95% CI (0.2.25–12.68), *p* < 0.005] and the UHR groups [2.96, 95% CI (0.44–5.48), *p* < 0.05, resp. 7.04, 95% CI (1.93–12.14), *p* < 0.005]. There was neither significant difference in low-frequency dipole strengths between HR and UHR groups nor between HC and at-risk-bip groups. For an illustration of the N20 differences in the cannabis non-users groups, see grand averages on Figure 2.

There was a significant effect of group on the maximum peak-to-peak amplitude of the early [*F*_(3,100)_ = 5.29, *p* < 0.005, *r* = 0.37] and of the late part of HFOs [*F*_(3,101)_ = 4.90, *p* < 0.005, *r* = 0.36]. *Post-hoc* tests revealed significantly lower early resp. late HFO amplitudes in the HR (0.60, 95% CI (0.05–1.14), *p* < 0.05 resp. 56, 95% CI (0.01–1.10), *p* < 0.05] and in the UHR groups (0.55, 95% CI (0.01–1.09), *p* < 0.05 resp. 63, 95% (0.09–1.17), *p* < 0.05] compared to the HC group. There was neither significant difference in high-frequency amplitudes between HR and UHR groups nor between HC and at-risk-bip groups. For details see Figure 1.

### Correlations of SEP-parameters with clinical symptoms

Over the whole group of cannabis non-users in both at-risk groups (*n* = 73), there was a significant negative correlation between N20 and the positive symptom scale of the PANSS, *r* = −0.26, *p* < 0.05, *r*^2^ = 0.068. This was paralleled by a trend toward a negative correlation between N20 and the PANSS positive factor of the 5-factor model of Citrome et al. (2011), *r* = −0.15, *p* = 0.063, *r*^2^ = 0.022, resp. Wallwork et al. (2012), *r* = −0.21, *p* = 0.076, *r*^2^ = 0.044. N20 correlated negatively with PANSS factor depression (Citrome et al., 2011), *r* = −0.29, *p* < 0.05, *r*^2^ = 0.086, paralleled by a trend with the factor depression of Wallwork et al. (2012), *r* = −0.23, *p* = 0.059, *r*^2^ = 0.051.

There was a significant positive correlation between latency of N20 and PANSS negative symptom scale, *r* = 0.25, *p* < 0.05, *r*^2^ = 0.061, paralleled by the correlation with PANSS negative factor of the 5-factor model of Wallwork et al. (2012), *r* = 0.25, *p* < 0.05, *r*^2^ = 0.061. N20 latency correlated with PANSS composite, *r* = −0.27, *p* < 0.05, *r*^2^ = 0.075. Furthermore, dipole strength of N20 correlated negatively with SPI-A subscale E “body perception disturbances,” *r* = −0.28, *p* < 0.05, *r*^2^ = 0.076. The latency of the early part of HFO correlated with SPIA subscale D “disturbances in experiencing the self and surroundings,” *r* = −0.29, *p* < 0.05, *r*^2^ = 0.086.

## Discussion

In this study, 25 subjects fulfilling the at-risk-bipolar criteria, 59 subjects the HR criteria for psychosis and 73 the UHR criteria for psychosis were compared with 45 controls, gender- and age-matched to the whole at-risk-group. We expected to already observe alterations of low- and high-frequency components of median nerve evoked SEPs in a population at risk for developing schizophrenia or bipolar disorder as these were reported in clinical populations. To our knowledge, this is the first study investigating SEPs in populations at risk for developing psychosis to date.

Firstly, among the whole sample (*n* = 202), compared with at-risk-bipolar subjects, a significant reduction of the mean strength of the N20 dipole was detected in both groups of subjects at risk for developing schizophrenia. The at-risk-bipolar group showed actually stronger N20 source activity than the healthy control group. Furthermore, both groups at risk for schizophrenia showed significant reduced amplitudes of the early and late part of the high-frequency signal (HFO) compared to the at-risk bipolar group. Both groups at risk for schizophrenia showed also reduced HFO amplitudes compared to the control group, but this difference reached significance only for the HR group. The at-risk bipolar group did not show such a reduction.

Secondly, among cannabis non-users, the amplitudes of the SEP parameters in the at-risk-bipolar group appeared to be similar to those of the control group, and not enhanced as in the cannabis users group. In both groups at risk for schizophrenia, the amplitudes of the SEP parameters were enhanced in cannabis users compared to non-users. Moreover, SEP amplitudes did not differ between cannabis users of the groups at risk for schizophrenia and non-users of the HC group. Rentzsch et al. (2011) reported similar effects of cannabis on MMN in schizophrenic patients. It was proposed that delta-9-tetracannabinol produces transient symptoms as well as behavioral and cognitive deficits resembling those seen in schizophrenia, increasing the clinical ratings of schizophrenia-like symptoms (Delisi, 2008). This may explain the observed effect of cannabis use in the present risk population. According to this, some subjects at risk for schizophrenia may not have shown risk symptoms if they did not have used cannabis. Another possible interpretation of the effects of cannabis postulates that cannabis may exhibit neuroprotective effects counteracting a putative neurotoxic process related to schizophrenia (Jockers-Scherubl et al., 2007; Potvin et al., 2008; Rentzsch et al., 2011). However, another explanation could be that risk subjects with comorbid cannabis use are less impaired compared to subjects without substance use.

Over the whole risk group of cannabis non-users, the above mentioned neurophysiological alterations were associated with impediments assessed on psychopathological level. Reduced low-frequency source activity were related to more positive symptoms as assessed by PANSS. Longer N20 latencies were associated with higher scores on PANSS negative and composite subscales. Reduced N20 source activity were also observed in subjects with more body perception disturbances (SPIA-E).

The findings of the present study are in accordance with other findings in individuals at risk for developing schizophrenia. At-risk individuals for schizophrenia showed significantly lower ERP amplitudes (P100, N170, N250) than healthy controls in a recognition task of facial affect (Wolwer et al., 2011). Sensory gating (P50 and N100) was already impaired in early stages of schizophrenia (at-risk, truly prodromal and first episode subjects), with most prominent impairments in chronic stages (Brockhaus-Dumke et al., 2008). Smaller P300 amplitudes were observed in high-risk individuals (Frommann et al., 2008). These ERP abnormalities are similar to those previously reported in manifest schizophrenia. Reduction of ERP amplitudes is a well-replicated finding in schizophrenia. The P50 amplitude and auditory sensory gating (McCarley et al., 1997; Adler et al., 1998), the amplitude of early P1 visual evoked potential (Koychev et al., 2012) as well as the amplitude of mismatch negativity (Atkinson et al., 2012) and reorienting negativity (Rissling et al., 2012) were shown to be diminished. Several ERPs have been discussed as vulnerability markers for schizophrenia (Van Der Stelt and Belger, 2007; Ladea and Prelipceanu, 2009). In contrast, neither of these early ERP components was reduced in bipolar disorder. Previous studies reported N100 and P200 components to be intact in manifest bipolar disorder (Muir et al., 1991; O'donnell et al., 2004). Furthermore, when similar neuroanatomic alterations were reported for both groups, these were mostly less severe in bipolar disorder than in schizophrenia (Maier et al., 2006; Yu et al., 2010).

As the amplitude reduction can be interpreted as a reduced sensory registration (Brockhaus-Dumke et al., 2008), this data is in line with the increasing body of evidence suggesting a gating deficit in schizophrenia. Moreover, in contrast to bipolar disorder, early sensory processing in schizophrenia is reduced already in at risk subjects, adding evidence to the assumption that specific sensory dysfunctions precede the onset of schizophrenia.

Under the assumption that late HFO represent the activities of GABAergic inhibitory interneurons in somatosensory cortex (Hashimoto et al., 1996; Ozaki et al., 2001; Ozaki and Hashimoto, 2005, 2011), the lower amplitudes of HFO in both schizophrenia-risk groups could point to a GABAergic dysfunction (Hasan et al., 2012). GABAergic interneurons provide both inhibitory and disinhibitory modulation of cortical and hippocampal circuits and contribute to the generation of oscillatory rhythms, discriminative information processing and gating of sensory information within the corticolimbic system (Benes and Berretta, 2001). Disruption of the balance between excitation and inhibition has been suggested to lead to gating defects that are related to cognitive impairment, as observed in schizophrenia (Marin, 2012). Glutaminergic neurons are the major excitatory pathways linking the cortex, limbic systems, and thalamus, regions that have been implicated in schizophrenia, with dysfunctional glutaminergic and related dopaminergic neurotransmission (Goff and Coyle, 2001). Recently, reduced thalamic glutamate levels were reported in people at risk for developing psychosis (Fusar-Poli et al., 2011). Kaufman et al. (2009) found no difference in brain GABA, glutamate or glutamine levels between patients with bipolar disorder and controls.

In contrast to the present findings, a previous SEP study in schizophrenia indicated higher N20 mean amplitude (Shagass et al., 1977; Lehtonen, 1981; Norra et al., 2004), and later HFO in patients compared to healthy controls (Norra et al., 2004). The different experimental groups, i.e., risk patients in the present sample and chronic patients in the study of Norra et al. (2004), may have contributed to the inconsistent findings between studies. First, the duration of illness had an effect on frequency of N20. In addition, all patients of Norra et al. (2004) were taking antipsychotic drugs before and during the study. This could have influenced the results, as other studies reported altered EP amplitudes (Straumanis et al., 1982; Duncan and Kaye, 1987; Duncan et al., 1987; Ford et al., 1994) and later ERP in medicated schizophrenic patients (Pfefferbaum et al., 1989).

Our study has methodological limitations. First, sample sizes were unequal. But as the data were normally distributed and the variances were roughly equal, we can assume that the power of the *F* statistic was not affected. Then, the UHR group was significantly younger than the other groups. A possible explanation for this could be given by the fact that individuals with higher risk to develop schizophrenia may fall ill younger. However, it appears unlikely that age did affect SEP parameters, primarily because age did not correlate with SEP parameters. Moreover, comparable SEP results were found for both the HR and the UHR group, while the HR group did not differ in mean age from the other groups. Additionally, more subjects from the UHR group were taking antipsychotic medication, compared to the other groups. The observed group effect might be confounded with the effect of medication. Another confounding effect may be that of alcohol and tobacco, as several subjects in a risk status for psychoses can be prone to use these substances. Finally, the correlations were not corrected for multiple testing, and given that the effect sizes are ranging from small to medium (*r* = 0.15–0.29) resp. the percentage of variance accounted for by the predictor variables is quite low (2.2–8.6%), they have to be interpreted with caution. At last, the present cross-sectional analysis limits the explanatory power of the data. Further longitudinal analysis of data from ZInEP is needed. Furthermore, the used method of dipole source analysis is not suitable for clinical practice. Whether a simple one-channel information would lead to comparable results requires further experimentation.

In summary, the findings of the present study suggest that the risk for schizophrenia, in contrast to bipolar disorder, may involve an impairment of early cerebral somatosensory processing. Neurophysiologic alterations in schizophrenia may precede the onset of initial psychotic episode and could therefore serve as indicator of vulnerability for developing schizophrenia. To our knowledge, this study is the largest investigation of somatosensory evoked potentials published to date, and the first in populations at risk for developing psychosis. The heterogeneity among studies and the lack of SEP studies comparing schizophrenia and bipolar disorder as well as the sparse studies comparing these populations on the risk level limit definitive conclusions from the literature to date.

### Conflict of interest statement

The authors declare that the research was conducted in the absence of any commercial or financial relationships that could be construed as a potential conflict of interest.

## References

[B1] Adler L. E. Olincy A. Waldo M. Harris J. G. Griffith J. Stevens K. (1998). Schizophrenia, sensory gating, and nicotinic receptors. Schizophr. Bull. 24, 189–202 10.1093/oxfordjournals.schbul.a033320 9613620

[B2] Alaerts M. Del-Favero J. (2009). Searching genetic risk factors for schizophrenia and bipolar disorder: learn from the past and back to the future. Hum. Mutat. 30, 1139–1152 10.1002/humu.21042 19626716

[B3] Allison T. McCarthy G. Wood C. C. Jones S. J. (1991). Potentials evoked in human and monkey cerebral cortex by stimulation of the median nerve. A review of scalp and intracranial recordings. Brain 114, 2465–2503 10.1093/brain/114.6.2465 1782527

[B4] Andreasen N. C. (1997). The role of the thalamus in schizophrenia. Can. J. Psychiatry 42, 27–33 9040920 10.1177/070674379704200104

[B5] Andreasen N. C. Arndt S. Swayze V. 2nd. Cizadlo T. Flaum M. O'leary D. (1994). Thalamic abnormalities in schizophrenia visualized through magnetic resonance image averaging. Science 266, 294–298 10.1126/science.7939669 7939669

[B6] Andreasen N. C. Pressler M. Nopoulos P. Miller D. Ho B.-C. (2010). Antipsychotic dose equivalents and dose-years: a standardized method for comparing exposure to different drugs. Biol. Psychiatry 67, 255–262 10.1016/j.biopsych.2009.08.040 19897178 PMC3677042

[B7] Angst J. Adolfsson R. Benazzi F. Gamma A. Hantouche E. Meyer T. D. (2005). The HCL-32: towards a self-assessment tool for hypomanic symptoms in outpatients. J. Affect. Disord. 88, 217–233 10.1016/j.jad.2005.05.011 16125784

[B8] Atkinson R. J. Michie P. T. Schall U. (2012). Duration mismatch negativity and P3a in first-episode psychosis and individuals at ultra-high risk of psychosis. Biol. Psychiatry 71, 98–104 10.1016/j.biopsych.2011.08.023 22000060

[B9] Benes F. M. Berretta S. (2001). GABAergic interneurons: implications for understanding schizophrenia and bipolar disorder. Neuropsychopharmacology 25, 1–27 10.1016/S0893-133X(01)00225-1 11377916

[B10] Braff D. L. (2010). Prepulse inhibition of the startle reflex: a window on the brain in schizophrenia, in Behavioral Neurobiology of Schizophrenia and its Treatment, ed Swerdlow N. R. (Berlin; Heidelberg: Springer), 349–371 10.1007/7854_2010_61 21312406

[B11] Brockhaus-Dumke A. Schultze-Lutter F. Mueller R. Tendolkar I. Bechdolf A. Pukrop R. (2008). Sensory gating in schizophrenia: P50 and N100 gating in antipsychotic-free subjects at risk, first-episode, and chronic patients. Biol. Psychiatry 64, 376–384 10.1016/j.biopsych.2008.02.006 18395700

[B12] Buchner H. Adams L. Muller A. Ludwig I. Knepper A. Thron A. (1995). Somatotopy of human hand somatosensory cortex revealed by dipole source analysis of early somatosensory evoked potentials and 3D-NMR tomography. Electroencephalogr. Clin. Neurophysiol. 96, 121–134 10.1016/0168-5597(94)00228-7 7535218

[B13] Buchsbaum M. S. Someya T. Teng C. Y. Abel L. Chin S. Najafi A. (1996). PET and MRI of the thalamus in never-medicated patients with schizophrenia. Am. J. Psychiatry 153, 191–199 8561198 10.1176/ajp.153.2.191

[B14] Buchsbaum M. S. Someya T. Wu J. C. Tang C. Y. Bunney W. E. (1997). Neuroimaging bipolar illness with positron emission tomography and magnetic resonance imaging. Psychiatr. Ann. 27, 489–495 10.3928/0048-5713-19970701-10

[B15] Caetano S. C. Sassi R. Brambilla P. Harenski K. Nicoletti M. Mallinger A. G. (2001). MRI study of thalamic volumes in bipolar and unipolar patients and healthy individuals. Psychiatry Res. 108, 161–168 10.1016/S0925-4927(01)00123-8 11756014

[B16] Carlsson M. Carlsson A. (1990). Interactions between glutamatergic and monoaminergic systems within the basal ganglia–implications for schizophrenia and Parkinson's disease. Trends Neurosci. 13, 272–276 10.1016/0166-2236(90)90108-M 1695402

[B17] Carroll C. A. Vohs J. L. O'donnell B. F. Shekhar A. Hetrick W. P. (2007). Sensorimotor gating in manic and mixed episode bipolar disorder. Bipolar Disord. 9, 221–229 10.1111/j.1399-5618.2007.00415.x 17430296

[B18] Citrome L. Meng X. Hochfeld M. (2011). Efficacy of iloperidone in schizophrenia: a PANSS five-factor analysis. Schizophr. Res. 131, 75–81 10.1016/j.schres.2011.05.018 21700430

[B19] Craddock N. Owen M. J. (2005). The beginning of the end for the Kraepelinian dichotomy. Br. J. Psychiatry 186, 364–366 10.1192/bjp.186.5.364 15863738

[B20] Crow T. J. (1986). The continuum of psychosis and its implication for the structure of the gene. Br. J. Psychiatry 149, 419–429 10.1192/bjp.149.4.419 3814925

[B21] Curio G. (2000). Linking 600-Hz “spikelike” EEG/MEG wavelets (“sigma-bursts”) to cellular substrates: concepts and caveats. J. Clin. Neurophysiol. 17, 377–396 10.1097/00004691-200007000-00004 11012041

[B22] Deicken R. F. Johnson C. Eliaz Y. Schuff N. (2000). Reduced concentrations of thalamic N-acetylaspartate in male patients with schizophrenia. Am. J. Psychiatry 157, 644–647 10.1176/appi.ajp.157.4.644 10739431

[B23] Delisi L. E. (2008). The effect of cannabis on the brain: can it cause brain anomalies that lead to increased risk for schizophrenia? Curr. Opin. Psychiatry 21, 140–150 10.1097/YCO.0b013e3282f51266 18332661 PMC4337025

[B24] Duncan C. C. Kaye W. H. (1987). Effects of clonidine on event-related potential measures of information processing. Electroencephalogr. Clin. Neurophysiol. Suppl. 40, 527–531 3480173

[B25] Duncan C. C. Perlstein W. M. Morihisa J. M. (1987). The P300 metric in schizophrenia: effects of probability and modality. Electroencephalogr. Clin. Neurophysiol. Suppl. 40, 670–674 2891497

[B26] Emerson R. G. Sgro J. A. Pedley T. A. Hauser W. A. (1988). State-dependent changes in the N20 component of the median nerve somatosensory evoked potential. Neurology 38, 64–68 10.1212/WNL.38.1.64 3336466

[B27] Ettinger U. Picchioni M. Landau S. Matsumoto K. Van Haren N. E. Marshall N. (2007). Magnetic resonance imaging of the thalamus and adhesio interthalamica in twins with schizophrenia. Arch. Gen. Psychiatry 64, 401–409 10.1001/archpsyc.64.4.401 17404117

[B28] Ford J. M. White P. M. Csernansky J. G. Faustman W. O. Roth W. T. Pfefferbaum A. (1994). ERPs in schizophrenia: effects of antipsychotic medication. Biol. Psychiatry 36, 153–170 10.1016/0006-3223(94)91221-1 7948453

[B29] Frommann I. Brinkmeyer J. Ruhrmann S. Hack E. Brockhaus-Dumke A. Bechdolf A. (2008). Auditory P300 in individuals clinically at risk for psychosis. Int. J. Psychophysiol. 70, 192–205 10.1016/j.ijpsycho.2008.07.003 18700155

[B30] Fusar-Poli P. Borgwardt S. Bechdolf A. Addington J. Riecher-Rössler A. Schultze-Lutter F. (2013). The psychosis high-risk state: a comprehensive state-of-the-art review. JAMA psychiatry 70, 107–120 10.1001/jamapsychiatry.2013.269 23165428 PMC4356506

[B31] Fusar-Poli P. Stone J. M. Broome M. R. Valli I. Mechelli A. McLean M. A. (2011). Thalamic glutamate levels as a predictor of cortical response during executive functioning in subjects at high risk for psychosis. Arch. Gen. Psychiatry 68, 881–890 10.1001/archgenpsychiatry.2011.46 21536967

[B32] Giakoumaki S. G. Roussos P. Rogdaki M. Karli C. Bitsios P. Frangou S. (2007). Evidence of disrupted prepulse inhibition in unaffected siblings of bipolar disorder patients. Biol. Psychiatry 62, 1418–1422 10.1016/j.biopsych.2006.12.002 17481589

[B33] Gobbele R. Waberski T. D. Kuelkens S. Sturm W. Curio G. Buchner H. (2000). Thalamic and cortical high-frequency (600 Hz) somatosensory-evoked potential (SEP) components are modulated by slight arousal changes in awake subjects. Exp. Brain Res. 133, 506–513 10.1007/s002210000435 10985685

[B34] Gobbele R. Waberski T. D. Simon H. Peters E. Klostermann F. Curio G. (2004). Different origins of low- and high-frequency components (600 Hz) of human somatosensory evoked potentials. Clin. Neurophysiol. 115, 927–937 10.1016/j.clinph.2003.11.009 15003775

[B35] Gobbele R. Waberski T. D. Thyerlei D. Thissen M. Darvas F. Klostermann F. (2003). Functional dissociation of a subcortical and cortical component of high-frequency oscillations in human somatosensory evoked potentials by motor interference. Neurosci. Lett. 350, 97–100 10.1016/S0304-3940(03)00877-2 12972162

[B36] Goff D. C. Coyle J. T. (2001). The emerging role of glutamate in the pathophysiology and treatment of schizophrenia. Am. J. Psychiatry 158, 1367–1377 10.1176/appi.ajp.158.9.1367 11532718

[B37] Gray C. M. McCormick D. A. (1996). Chattering cells: superficial pyramidal neurons contributing to the generation of synchronous oscillations in the visual cortex. Science 274, 109–113 10.1126/science.274.5284.109 8810245

[B38] Gur R. E. Maany V. Mozley P. D. Swanson C. Bilker W. Gur R. C. (1998). Subcortical MRI volumes in neuroleptic-naive and treated patients with schizophrenia. Am. J. Psychiatry 155, 1711–1717 9842780 10.1176/ajp.155.12.1711

[B39] Gururajan A. Manning E. E. Klug M. Van Den Buuse M. (2012). Drugs of abuse and increased risk of psychosis development. Aust. N. Z. J. Psychiatry 46, 1120–1135 10.1177/0004867412455232 22833579

[B40] Halboni P. Kaminski R. Gobbele R. Zuchner S. Waberski T. D. Herrmann C. S. (2000). Sleep stage dependant changes of the high-frequency part of the somatosensory evoked potentials at the thalamus and cortex. Clin. Neurophysiol. 111, 2277–2284 10.1016/S1388-2457(00)00473-9 11090782

[B41] Hasan A. Wobrock T. Grefkes C. Labusga M. Levold K. Schneider-Axmann T. (2012). Deficient inhibitory cortical networks in antipsychotic-naive subjects at risk of developing first-episode psychosis and first-episode schizophrenia patients: a cross-sectional study. Biol. Psychiatry 72, 744–751 10.1016/j.biopsych.2012.03.005 22502988

[B42] Hashimoto I. Kimura T. Fukushima T. Iguchi Y. Saito Y. Terasaki O. (1999). Reciprocal modulation of somatosensory evoked N20m primary response and high-frequency oscillations by interference stimulation. Clin. Neurophysiol. 110, 1445–1451 10.1016/S1388-2457(99)00083-8 10454280

[B43] Hashimoto I. Mashiko T. Imada T. (1996). Somatic evoked high-frequency magnetic oscillations reflect activity of inhibitory interneurons in the human somatosensory cortex. Electroencephalogr. Clin. Neurophysiol. 100, 189–203 10.1016/0168-5597(95)00244-8 8681860

[B44] Hazlett E. A. Buchsbaum M. S. Byne W. Wei T. C. Spiegel-Cohen J. Geneve C. (1999). Three-dimensional analysis with MRI and PET of the size, shape, and function of the thalamus in the schizophrenia spectrum. Am. J. Psychiatry 156, 1190–1199 10450259 10.1176/ajp.156.8.1190

[B45] Holcomb H. H. Cascella N. G. Thaker G. K. Medoff D. R. Dannals R. F. Tamminga C. A. (1996). Functional sites of neuroleptic drug action in the human brain: PET/FDG studies with and without haloperidol. Am. J. Psychiatry 153, 41–49 8540590 10.1176/ajp.153.1.41

[B46] Jockers-Scherubl M. C. Wolf T. Radzei N. Schlattmann P. Rentzsch J. Gomez-Carrillo De Castro A. (2007). Cannabis induces different cognitive changes in schizophrenic patients and in healthy controls. Prog. Neuropsychopharmacol. Biol. Psychiatry 31, 1054–1063 10.1016/j.pnpbp.2007.03.006 17482741

[B47] Jones E. G. (1997). Cortical development and thalamic pathology in schizophrenia. Schizophr. Bull. 23, 483–501 10.1093/schbul/23.3.483 9327511

[B48] Kaufman R. E. Ostacher M. J. Marks E. H. Simon N. M. Sachs G. S. Jensen J. E. (2009). Brain GABA levels in patients with bipolar disorder. Prog. Neuropsychopharmacol. Biol. Psychiatry 33, 427–434 10.1016/j.pnpbp.2008.12.025 19171176

[B49] Kawohl W. Rössler W. (2008). Cannabis und Schizophrenie: Neue Erkenntnisse in einer alten Debatte. Neuropsychiatrie 22, 223–229 19080993

[B50] Kay S. R. Fiszbein A. Opfer L. A. (1987). The positive and negative syndrome scale (PANSS) for schizophrenia. Schizophr. Bull. 13, 261–276 10.1093/schbul/13.2.261 3616518

[B51] Kim J. J. Mohamed S. Andreasen N. C. O'leary D. S. Watkins G. L. Boles Ponto L. L. (2000). Regional neural dysfunctions in chronic schizophrenia studied with positron emission tomography. Am. J. Psychiatry 157, 542–548 10.1176/appi.ajp.157.4.542 10739412

[B52] Klosterkötter J. Schultze-Lutter F. Bechdolf A. Ruhrmann S. (2011). Prediction and prevention of schizophrenia: what has been achieved and where to go next? World Psychiatry 10, 165–174 10.1002/j.2051-5545.2011.tb00044.x 21991266 PMC3190243

[B53] Klostermann F. Nolte G. Curio G. (1999). Multiple generators of 600 Hz wavelets in human SEP unmasked by varying stimulus rates. Neuroreport 10, 1625–1629 10.1097/00001756-199906030-00001 10501547

[B54] Klostermann F. Nolte G. Losch F. Curio G. (1998). Differential recruitment of high frequency wavelets (600 Hz) and primary cortical response (N20) in human median nerve somatosensory evoked potentials. Neurosci. Lett. 256, 101–104 10.1016/S0304-3940(98)00773-3 9853713

[B55] Koychev I. El-Deredy W. Mukherjee T. Haenschel C. Deakin J. F. (2012). Core dysfunction in schizophrenia: electrophysiology trait biomarkers. Acta Psychiatr. Scand. 126, 59–71 10.1111/j.1600-0447.2012.01849.x 22384856

[B56] Kurnianingsih Y. A. Kuswanto C. N. McIntyre R. S. Qiu A. Ho B. C. Sim K. (2011). Neurocognitive-genetic and neuroimaging-genetic research paradigms in schizophrenia and bipolar disorder. J. Neural Transm. 118, 1621–1639 10.1007/s00702-011-0672-z 21688113

[B57] Ladea M. Prelipceanu D. (2009). Markers of vulnerability in schizophrenia. J. Med. Life 2, 155–164 20108534 PMC3018975

[B58] Lehtonen J. (1981). Somatosensory evoked potentials and the psychology of chronic schizophrenia. An integrative view. J. Nerv. Ment. Dis. 169, 256–258 10.1097/00005053-198104000-00010 7217933

[B59] Maier W. Zobel A. Wagner M. (2006). Schizophrenia and bipolar disorder: differences and overlaps. Curr. Opin. Psychiatry 19, 165–170 10.1097/01.yco.0000214342.52249.82 16612197

[B60] Mamah D. Wang L. Csernansky J. G. Rice J. P. Smith M. Barch D. M. (2010). Morphometry of the hippocampus and amygdala in bipolar disorder and schizophrenia. Bipolar Disord. 12, 341–343 10.1111/j.1399-5618.2010.00802.x 20565442 PMC4461218

[B61] Marin O. (2012). Interneuron dysfunction in psychiatric disorders. Nat. Rev. Neurosci. 13, 107–120 10.1038/nrn3155 22251963

[B62] McCarley R. W. O'donnell B. F. Niznikiewicz M. A. Salisbury D. F. Potts G. F. Hirayasu Y. (1997). Update on electrophysiology in schizophrenia. Int. Rev. Psychiatr. 9, 373 10.1080/09540269775240

[B63] McCormick D. A. Bal T. (1994). Sensory gating mechanisms of the thalamus. Curr. Opin. Neurobiol. 4, 550–556 10.1016/0959-4388(94)90056-6 7812144

[B64] McDonald C. Bullmore E. T. Sham P. C. Chitnis X. Wickham H. Bramon E. (2004). Association of genetic risks for schizophrenia and bipolar disorder with specific and generic brain structural endophenotypes. Arch. Gen. Psychiatry 61, 974–984 10.1001/archpsyc.61.10.974 15466670

[B65] McGlashan T. H. Miller T. J. Woods S. W. Rosen J. L. Hoffman R. E. Davidson L. (2001). Structured Interview for Prodromal Syndromes. New Haven, CT: PRIME Research Clinic; Yale School of Medicine

[B66] Muir W. J. St Clair D. M. Blackwood D. H. (1991). Long-latency auditory event-related potentials in schizophrenia and in bipolar and unipolar affective disorder. Psychol. Med. 21, 867–879 10.1017/S003329170002986X 1780401

[B67] Murray R. M. Sham P. Van Os J. Zanelli J. Cannon M. McDonald C. (2004). A developmental model for similarities and dissimilarities between schizophrenia and bipolar disorder. Schizophr. Res. 71, 405–416 10.1016/j.schres.2004.03.002 15474912

[B68] Norra C. Waberski T. D. Kawohl W. Kunert H. J. Hock D. Gobbele R. (2004). High-frequency somatosensory thalamocortical oscillations and psychopathology in schizophrenia. Neuropsychobiology 49, 71–80 10.1159/000076413 14981337

[B69] O'donnell B. F. Vohs J. L. Hetrick W. P. Carroll C. A. Shekhar A. (2004). Auditory event-related potential abnormalities in bipolar disorder and schizophrenia. Int. J. Psychophysiol. 53, 45–55 10.1016/j.ijpsycho.2004.02.001 15172135

[B70] Ozaki I. Hashimoto I. (2005). Neural mechanisms of the ultrafast activities. Clin. EEG Neurosci. 36, 271–277 16296444 10.1177/155005940503600406

[B71] Ozaki I. Hashimoto I. (2011). Exploring the physiology and function of high-frequency oscillations (HFOs) from the somatosensory cortex. Clin. Neurophysiol. 122, 1908–1923 10.1016/j.clinph.2011.05.023 21724458

[B72] Ozaki I. Yaegashi Y. Kimura T. Baba M. Matsunaga M. Hashimoto I. (2001). Dipole orientation differs between high frequency oscillations and N20m current sources in human somatosensory evoked magnetic fields to median nerve stimulation. Neurosci. Lett. 310, 41–44 10.1016/S0304-3940(01)02090-0 11524153

[B73] Perry W. Minassian A. Feifel D. Braff D. L. (2001). Sensorimotor gating deficits in bipolar disorder patients with acute psychotic mania. Biol. Psychiatry 50, 418–424 10.1016/S0006-3223(01)01184-2 11566158

[B74] Pfefferbaum A. Ford J. M. White P. M. Roth W. T. (1989). P3 in schizophrenia is affected by stimulus modality, response requirements, medication status, and negative symptoms. Arch. Gen. Psychiatry 46, 1035–1044 10.1001/archpsyc.1989.01810110077011 2573328

[B75] Portas C. M. Goldstein J. M. Shenton M. E. Hokama H. H. Wible C. G. Fischer I. (1998). Volumetric evaluation of the thalamus in schizophrenic male patients using magnetic resonance imaging. Biol. Psychiatry 43, 649–659 10.1016/S0006-3223(97)00339-9 9582998

[B76] Potvin S. Joyal C. C. Pelletier J. Stip E. (2008). Contradictory cognitive capacities among substance-abusing patients with schizophrenia: a meta-analysis. Schizophr. Res. 100, 242–251 10.1016/j.schres.2007.04.022 17614260

[B77] Redpath H. L. Cooper D. Lawrie S. M. (2013). Imaging symptoms and syndromes: similarities and differences between schizophrenia and bipolar disorder. Biol. Psychiatry 73, 495–496 10.1016/j.biopsych.2013.01.015 23438631

[B78] Rentzsch J. Buntebart E. Stadelmeier A. Gallinat J. Jockers-Scherübl M. C. (2011). Differential effects of chronic cannabis use on preattentional cognitive functioning in abstinent schizophrenic patients and healthy subjects. Schizophr. Res. 130, 222–227 10.1016/j.schres.2011.05.011 21624823

[B79] Rissling A. J. Braff D. L. Swerdlow N. R. Hellemann G. Rassovsky Y. Sprock J. (2012). Disentangling early sensory information processing deficits in schizophrenia. Clin. Neurophysiol. 123, 1942–1949 10.1016/j.clinph.2012.02.079 22608970 PMC3436955

[B80] Ritter P. Freyer F. Curio G. Villringer A. (2008). High-frequency (600 Hz) population spikes in human EEG delineate thalamic and cortical fMRI activation sites. Neuroimage 42, 483–490 10.1016/j.neuroimage.2008.05.026 18586526

[B81] Roser P. Vollenweider F. X. Kawohl W. (2010). Potential antipsychotic properties of central cannabinoid (CB1) receptor antagonists. World J. Biol. Psychiatry 11, 208–219 10.3109/15622970801908047 20218784

[B82] Schultze-Lutter F. Addington J. Ruhrmann S. Klosterkötter J. (2007). Schizophrenia Proneness Instrument, Adult Version (SPI-A). Rome: Giovanni Fioriti

[B83] Schultze-Lutter F. Koch E. (2010). Schizophrenia Pronennes Instrument, Child & Youth Version (SPI-CY). Rome: Giovanni Fioriti

[B84] Shagass C. Straumanis J. J. Jr. Roemer R. A. Amadeo M. (1977). Evoked potentials of schizophrenics in several sensory modalities. Biol. Psychiatry 12, 221–235 870094

[B85] Sheehan D. V. Lecrubier Y. Sheehan K. H. Amorim P. Janavs J. Weiller E. (1998). The Mini-International Neuropsychiatric Interview (M.I.N.I.): the development and validation of a structured diagnostic psychiatric interview for DSM-IV and ICD-10. J. Clin. Psychiatry 59 Suppl. 20, 22–33; quiz 34–57. 9881538

[B86] Siegel B. V. Jr. Buchsbaum M. S. Bunney W. E. Jr. Gottschalk L. A. Haier R. J. Lohr J. B. (1993). Cortical-striatal-thalamic circuits and brain glucose metabolic activity in 70 unmedicated male schizophrenic patients. Am. J. Psychiatry 150, 1325–1336 8352343 10.1176/ajp.150.9.1325

[B87] Soares J. C. Mann J. J. (1997). The functional neuroanatomy of mood disorders. J. Psychiatr. Res. 31, 393–432 10.1016/S0022-3956(97)00016-2 9352470

[B88] Staal W. G. Hulshoff Pol H. E. Schnack H. Van Der Schot A. C. Kahn R. S. (1998). Partial volume decrease of the thalamus in relatives of patients with schizophrenia. Am. J. Psychiatry 155, 1784–1786 9842796 10.1176/ajp.155.12.1784

[B89] Straumanis J. J. Shagass C. Roemer R. A. (1982). Influence of antipsychotic and antidepressant drugs on evoked potential correlates of psychosis. Biol. Psychiatry 17, 1101–1122 6129004

[B90] Szechtman H. Nahmias C. Garnett E. S. Firnau G. Brown G. M. Kaplan R. D. (1988). Effect of neuroleptics on altered cerebral glucose metabolism in schizophrenia. Arch. Gen. Psychiatry 45, 523–532 10.1001/archpsyc.1988.01800300019002 2897836

[B91] Thaker G. (2008). Psychosis endophenotypes in schizophrenia and bipolar disorder. Schizophr. Bull. 34, 720–721 10.1093/schbul/sbn055 18503040 PMC2632455

[B92] Van Der Stelt O. Belger A. (2007). Application of electroencephalography to the study of cognitive and brain functions in schizophrenia. Schizophr. Bull. 33, 955–970 10.1093/schbul/sbm016 17363840 PMC2632335

[B93] Waberski T. D. Norra C. Kawohl W. Thyerlei D. Hock D. Klostermann F. (2004). Electrophysiological evidence for altered early cerebral somatosensory signal processing in schizophrenia. Psychophysiology 41, 361–366 10.1111/1469-8986.2004.00163.x 15102120

[B94] Wallwork R. S. Fortgang R. Hashimoto R. Weinberger D. R. Dickinson D. (2012). Searching for a consensus five-factor model of the positive and negative syndrome scale for schizophrenia. Schizophr. Res. 137, 246–250 10.1016/j.schres.2012.01.031 22356801 PMC3351536

[B95] Whalley H. C. Papmeyer M. Sprooten E. Lawrie S. M. Sussmann J. E. McIntosh A. M. (2012). Review of functional magnetic resonance imaging studies comparing bipolar disorder and schizophrenia. Bipolar Disord. 14, 411–431 10.1111/j.1399-5618.2012.01016.x 22631622

[B96] Williams J. B. (1988). A structured interview guide for the hamilton depression rating scale. Arch. Gen. Psychiatry 45, 742–747 10.1001/archpsyc.1988.01800320058007 3395203

[B97] Wolwer W. Brinkmeyer J. Stroth S. Streit M. Bechdolf A. Ruhrmann S. (2011). Neurophysiological correlates of impaired facial affect recognition in individuals at risk for schizophrenia. Schizophr. Bull. 38, 1021–1029 10.1093/schbul/sbr013 21402721 PMC3446235

[B98] Woodward N. D. Karbasforoushan H. Heckers S. (2012). Thalamocortical dysconnectivity in schizophrenia. Am. J. Psychiatry 169, 1092–1099 10.1176/appi.ajp.2012.12010056 23032387 PMC3810300

[B99] Yamada T. Kameyama S. Fuchigami Y. Nakazumi Y. Dickins Q. S. Kimura J. (1988). Changes of short latency somatosensory evoked potential in sleep. Electroencephalogr. Clin. Neurophysiol. 70, 126–136 10.1016/0013-4694(88)90113-7 2456191

[B100] Yu K. Cheung C. Leung M. Li Q. Chua S. McAlonan G. (2010). Are bipolar disorder and schizophrenia neuroanatomically distinct? An anatomical likelihood meta-analysis. Front. Hum. Neurosci. 4:189 10.3389/fnhum.2010.00189 21103008 PMC2987512

